# An adhesive elastomeric supramolecular polyurethane healable at body temperature†

**DOI:** 10.1039/c5sc04864h

**Published:** 2016-03-15

**Authors:** Antonio Feula, Xuegang Tang, Ioannis Giannakopoulos, Ann M. Chippindale, Ian W. Hamley, Francesca Greco, C. Paul Buckley, Clive R. Siviour, Wayne Hayes

**Affiliations:** a Department of Chemistry, University of Reading Whiteknights Reading RG6 6AD UK w.c.hayes@reading.ac.uk +44 (0)118-378-6331; b Department of Engineering Science, Oxford University Parks Road Oxford OX1 3PJ UK; c Reading School of Pharmacy, University of Reading Whiteknights Reading RG6 6AD UK

## Abstract

In this paper, we report the synthesis and healing ability of a non-cytotoxic supramolecular polyurethane network whose mechanical properties can be recovered efficiently (>99%) at the temperature of the human body (37 °C). Rheological analysis revealed an acceleration in the drop of the storage modulus above 37 °C, on account of the dissociation of the supramolecular polyurethane network, and this decrease in viscosity enables the efficient recovery of the mechanical properties. Microscopic and mechanical characterisation has shown that this material is able to recover mechanical properties across a damage site with minimal contact required between the interfaces and also demonstrated that the mechanical properties improved when compared to other low temperature healing elastomers or gel-like materials. The supramolecular polyurethane was found to be non-toxic in a cytotoxicity assay carried out in human skin fibroblasts (cell viability > 94% and non-significantly different compared to the untreated control). This supramolecular network material also exhibited excellent adhesion to pig skin and could be healed completely *in situ* post damage indicating that biomedical applications could be targeted, such as artificial skin or wound dressings with supramolecular materials of this type.

## Abstract 2

We report a non-cytotoxic supramolecular polyurethane network whose mechanical properties can be recovered efficiently (>99%) at body temperature.

## Graphical abstract

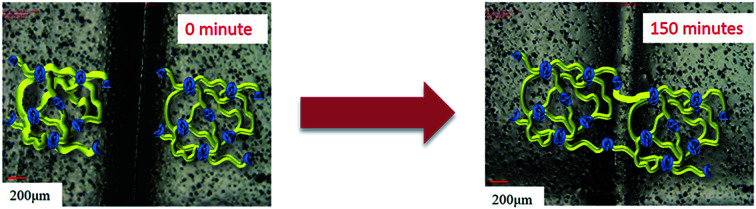

## Introduction

Materials capable of self-repair offer attractive advantages in many applications, especially in terms of performance and longevity.^1–6^ In recent years, polymers have been reported that can heal when external stimuli (such as heat,^7–13^ pressure,^14,15^ light^16–18^) are applied to damaged sites.^19,20^ Potential applications for healable materials within modern society include paints,^21–23^ aerospace composites,^24^ regenerative medicine,^25^ (in particular artificial skin^26–28^) and plastic surgery.^29–31^ Successful approaches to healable polymer networks that have been reported to date include encapsulated-monomer systems,^24,32–34^ reversible covalent bond formation,^15,35,36^ utilisation of irreversible covalent bond processes^37^ and more recently supramolecular self-assembly.^38–40^ In the latter approach, network formation is facilitated^40^ by a combination of non-covalent bond association (hydrogen bonds,^39,41–44^ electrostatic interactions,^45^ aromatic π–π stacking interactions^7–10,46^ or dynamic metal–ligand bonds^36,47^) and phase separation between the polar end-groups and apolar polymer chains, which serves to strengthen the end-groups aggregation, resulting in enhancement of the supramolecular interactions.^10,48^ The weak nature of non-covalent interactions permits the materials to possess thermo-responsive and thermo-reversible properties, thereby delivering dramatic viscosity changes over well-defined and tuneable temperature ranges. These addressable and tuneable characteristics^41,49^ are highly desirable in both bulk commodity and value-added applications, such as adhesives,^50^ shape-memory materials,^51,52^ healable coatings^10^ and impact-resistant structures (*e.g.* protection for mobile electronics). An important class of supramolecular polymers, which have been developed in the last decade, are polyurethanes. Supramolecular polyurethanes (SPUs)^53,54^ are synthesised *via* reaction of diols or polyols with polyisocyanates and alcohols or amines.^55^ The physical properties of SPUs have been shown to directly correlate^41^ to the nature of the hydrogen bond receptors that are generated by the reaction of isocyanate end groups and alcohols or amines.^48,56–58^

The generation of synthetic materials able to mimic human skin or that are suited for rapid wound isolation is a notable challenge in the biomedical industry.^59^ Supramolecular polymers have already been employed in biomedicine as biocompatible thermoplastic elastomers^38,60^ and acrylic copolymers utilised in wound dressings in the form of commercially available spray plasters (*e.g.* Elastoplast®, OpSITE™ and TCP®).^61–64^ Whilst elastomeric, these acrylic-based materials have not been described as healable in nature and thus new skin coatings whose properties offer the ability to repair physical damage *in situ* by taking advantage of the thermal energy provided by the host but without the requirement of a stimulus such as electrical current^65,66^ represent a significant practical advancement. In this paper, we report the synthesis and healing ability of a SPU whose mechanical properties can be recovered at the temperature of the human body (37 °C). In order to demonstrate the potential use of this SPU system in a biomedical setting, we also reveal that films of this material adhere to pig skin and can be healed *in situ* post damage.^67^

## Results and discussion

**Fig. 1:**
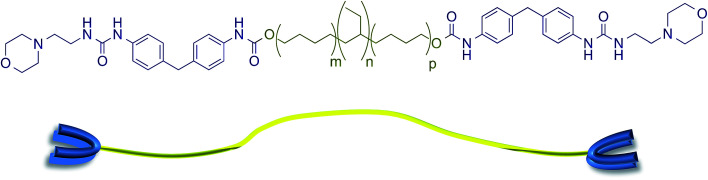
The supramolecular polyurethane 1 featuring urea morpholine end groups and a schematic representation of this polymer.

The supramolecular polyurethane 1 was synthesized^41,42,48^ using an established two-step process. Firstly, a hydrophobic and elastomeric diol, Krasol™ HLBH-P2000, was reacted with methylene diphenyl diisocyanate (MDI) at 80 °C for three hours to afford a prepolymer featuring isocyanate end-groups. 4-(2-Aminoethyl)morpholine was then added to the prepolymer to install the receptor end-groups *via* urea bond formation and afford the desired polyurethane 1 in a yield of 93% (see Fig. 1 plus ESI Fig. S1–S3† for spectroscopic and thermal data). Chain extension in polyurethane 1 was minimal, ^1^H NMR spectroscopic analysis revealed a ratio of 1 : 1 for the integrals of the proton resonances of the urethane and urea groups of the end-capping units, consistent with the feed ratios used in the prepolymer and end-capping steps. GPC analysis (THF, room temperature) revealed a material with *M*_n_ and *M*_w_ values of 4097 and 4287, respectively.

**Fig. 2:**
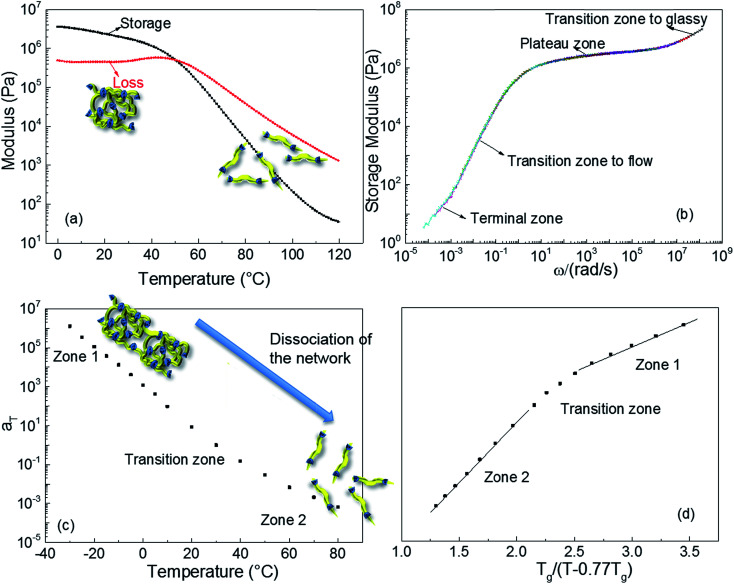
The rheological behaviour of polyurethane 1. (a) Temperature sweep at 5 Hz; (b) master curve at a reference temperature of 30 °C, different colours corresponding to different temperatures; (c) time–temperature shift factor *a*_T_ of polyurethane 1 as a function of temperature; (d) plot of *a*_T_ for polyurethane 1 as a function of temperature normalized to *T*_g_.

Dynamic rheological testing was employed to characterise the viscoelastic properties of the polyurethane 1. Temperature and frequency sweeps were performed using an Anton-Paar Physica MCR301 Rheometer, in oscillatory shear. In the low temperatures regime (from 0 to 35 °C, Fig. 2a), the elastic rubbery character clearly dominates the properties; there is a gradual decrease of storage modulus and concomitant constant loss modulus with increasing temperature. However, the drop of the storage modulus accelerates above 37 °C, owing to the dissociation of the supramolecular polyurethane network formed,^10^ and viscous behaviour governs the properties of polyurethane 1 at the temperature of 50 °C and above. The master curve shown in Fig. 2b was constructed by manual shifting of isothermal frequency sweep data (the raw data are available in the ESI, Fig. S4†).^68^ The overlapping of data obtained at different temperatures indicates that time/temperature superposition analysis was applied successfully to the rheological data for polyurethane 1. The resultant master curve shows the highly rate dependent behaviour of polyurethane 1 with a typical terminal zone, transition zone to flow, plateau zone (rubbery), and transition zone to glassy behaviour, as indicated in Fig. 2b, over the full investigated frequency regime.

Fig. 2c shows the shift factors, *a*_T_, used to produce the master curve, as a function of temperature. It is observed that *a*_T_ changes by over 9 orders of magnitude between −30 and +80 °C. This large change in *a*_T_ over a small, readily accessible temperature range is consistent with dissociation of the supramolecular network, leading to a large drop in viscosity that thereby facilitates healing of damage sites. This dramatic change is attributed to additional relaxation processes, which do not occur in amorphous covalently bonded polymers, and to the disengagement of the supramolecular (π–π stacking or hydrogen-bonding) interactions. For amorphous, covalently bonded polymers, there is a linear relationship between *a*_T_ and normalised temperature, but recent research shows that this trend is not observed in the case of supramolecular polymers.^69,70^ The behaviour of polyurethane 1, shown in Fig. 2d, is consistent with other supramolecular polymer systems:^8,10,68–70^ two linear zones are evident, with a transition associated with the dissociation of the network, but with a lower transition temperature than observed in previously reported materials. Importantly, although the mechanical response of polyurethane 1 is comparable to other supramolecular polymers and blends, the temperature at which the intermolecular interactions disengage is close to body temperature.

**Fig. 3:**
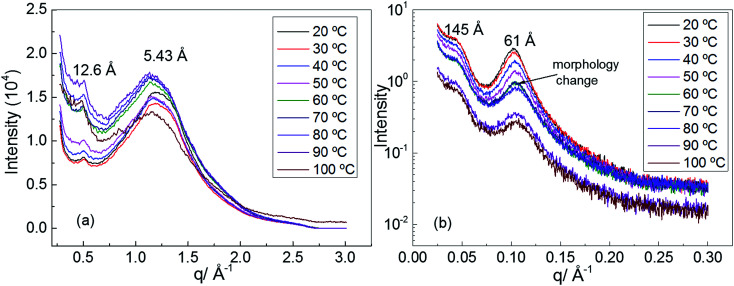
(a) Wide angle X-ray scattering (WAXS) of polyurethane 1, (b) small angle X-ray scattering (SAXS) of polyurethane 1.

In order to understand the morphology of polyurethane 1, variable temperature (20 to 100 °C) wide angle X-ray scattering (WAXS) and small angle X-ray scattering (SAXS) analyses were conducted. The WAXS scattering pattern shows a lattice spacing of 5.43 Å corresponding to the stacking of the urea moieties (Fig. 3a).^71^ It is interesting to note that with increasing temperature the lattice spacing becomes consistently less sharp, suggesting that the hydrogen bonding interactions between the urea moieties of polyurethane 1 are being disrupted. In particular, the change of the morphology is evident at a temperature of 60 °C in accordance with the rheology data.

Two Bragg peaks (61 and 145 Å) are evident in the SAXS profile (Fig. 3b), suggesting a microphase-separated morphology arising from the immiscibility of the hard hydrogen bonding end-groups with the soft polymer backbone. In addition, in the SAXS profile a drastic change in the morphology is evident at a temperature of 60 °C.

**Fig. 4:**
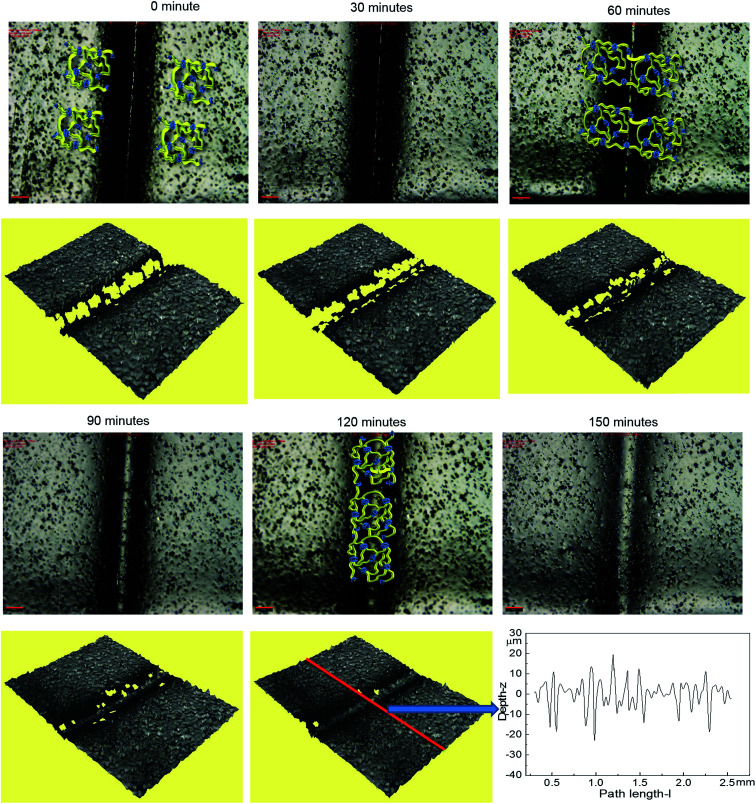
The dynamics of the healing for polyurethane 1 at the temperature of 37 °C (2D and 3D microscopy). The roughness profile is from the middle of the whole sample across the crack horizontally as indicated in the figure by the red line. The scale bar in the picture is 200 μm. Cartoons have been added to 0, 60 and 120 minute images to illustrate the healing process.

Optical microscopy was first used to probe the healing ability of this supramolecular material. In the light of the rheological data polyurethane 1 was exposed to a temperature of 37 °C and the dynamics of the healing process at this temperature was monitored. A sample was cut along the centre, transverse to its long axis with a razor, and then positioned with the cut edges in close contact. Fig. 4 shows two dimensional microscopy images, which reveal that the material surrounding the cut flows into the damage site upon heat treatment and after 120 minutes, the material becomes essentially homogenous with the position of the cut barely visible. Furthermore, the three dimensional surface profilometry images (Fig. 4) revealed the same behaviour and the surface rough profile shows that the roughness around the cut area is both qualitatively (*i.e.* visibly) and quantitatively comparable to the other areas in the surface of the sample, indicating a fully topological recovery of the cut interface.

**Fig. 5:**
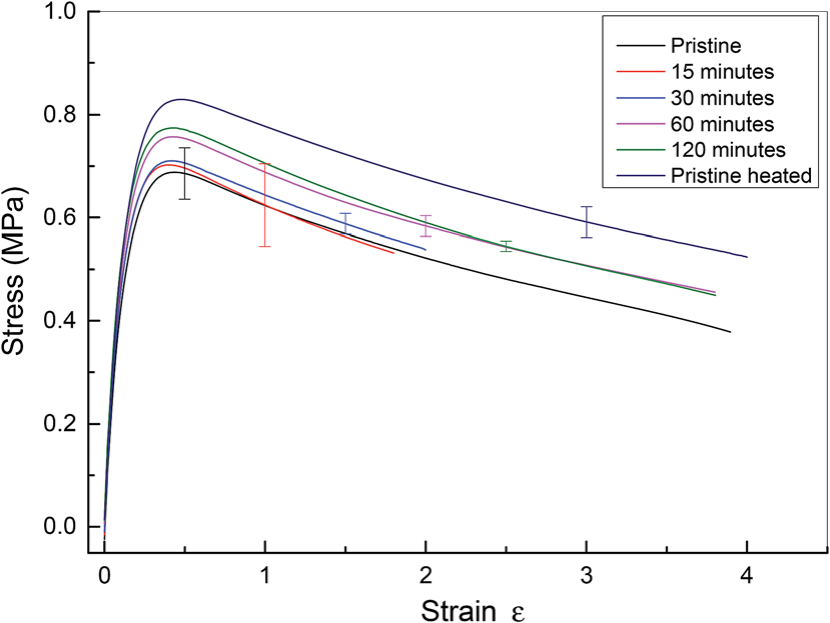
Mean stress–strain curve for pristine and healed polyurethane 1. Pristine material has no heat treatment. Data from cut and healed material are presented after healing for 15, 30, 60 and 120 minutes. Finally, data are presented for pristine material heated to 37 °C for 120 minutes. Data from all specimens are shown in the ESI, see Fig. S5.† Note that in this figure, the end of the stress–strain curve does not indicate specimen failure.

The microscopy data reveal that the physical integrity of polyurethane 1 can be recovered within two hours at a temperature of 37 °C. To examine the recovery of the mechanical properties after healing, tensile tests were performed on specimens *ca.* 0.5 mm thick, 40 mm long and 5 mm wide. For the healed samples, the two cut edges were positioned in contact, but not overlapped, as described in previous studies.^7–10^ Experiments were performed on pristine materials and specimens healed for different time periods at 37 °C. Pristine material heated to 37 °C for 120 minutes was also tested. Mean stress–strain curves with error bars are shown in Fig. 5, and the corresponding mechanical properties calculated from the individual stress–strain curves (see Fig. S5†) are shown in Table 1. It should be noted that in these experiments, the strain was calculated using digital image correlation on images of the specimen surface. The tensile modulus was calculated from the slope of the stress–strain curve between 0 and 4% strain. It was observed that thermal annealing improved the mechanical properties of polyurethane 1, as revealed by the apparent modulus of 6.32 MPa, yield strength of 0.68 MPa for pristine polyurethane 1 and 6.81 MPa and 0.82 MPa, respectively, for heat treated material after 120 minutes at 37 °C, indicating the reorganisation of the supramolecular network to attain the thermodynamic minimum. For the healed samples, it was observed that the mechanical properties recovered steadily and after 60 minutes, the mechanical performance was comparable to uncut pristine samples (apparent modulus: 6.72 MPa *vs.* 6.81 MPa, yield strength: 0.76 MPa *vs.* 0.82 MPa, elongation at break: 431% *vs.* 427%, and energy absorbed: 243 MPa *vs.* 269 MPa for sample healed after 60 minutes and heat treated polyurethane 1 after 120 minutes, respectively). Therefore, it can be concluded that polyurethane 1 can completely recover all the mechanical performance after healing at a temperature of 37 °C for 60 minutes.

**Table 1:** Mechanical properties from tensile testing of polyurethane 1 with different healing times; means and (standard deviations) from at least four samples. Names correspond to Fig. 5

Sample	Tensile modulus^a^ (MPa)	Yield strength (MPa)	Elongation at break (%)	Energy absorbed (MPa)
Pristine	6.32 ± 0.36	0.68 ± 0.05	405 ± 47	207 ± 23
15 minutes	6.38 ± 0.34	0.66 ± 0.08	234 ± 147	124 ± 74
30 minutes	6.64 ± 0.34	0.72 ± 0.02	222 ± 137	129 ± 62
60 minutes	6.72 ± 0.38	0.76 ± 0.02	431 ± 40	243 ± 18
120 minutes	6.93 ± 0.27	0.77 ± 0.01	432 ± 45	248 ± 21
Pristine heated	6.81 ± 0.20	0.82 ± 0.03	427 ± 217	269 ± 126

a This modulus was calculated using forces measured by the mechanical testing machine and local strains measured using an optical technique (digital image correlation) between strains of 0 and 3.5%. The value is therefore a linear approximation to the true, non-linear, polymer behaviour.

**Fig. 6:**
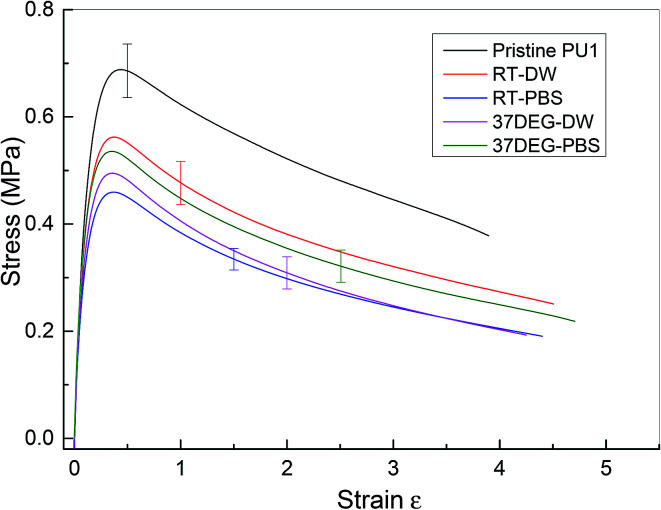
Mean stress–strain curve for pristine and polyurethane 1 at different physiological conditions. (Pristine PU1) pristine polyurethane 1, (RT-DW) at room temperature in distilled water for 48 hours, (RT-PBS) at room temperature in PBS solution for 48 hours, (37DEG-DW) at 37 °C in distilled water for 12 hours and (37DEG-PBS) at 37 °C in PBS solution for 12 hours. Samples were wiped dry before testing.

The investigation of the healing properties of polyurethane 1 demonstrates that it can recover its mechanical properties fully at body temperature after 60 minutes, which suggests that it has the potential to be used as a biomedical material, such as artificial skin and as an adhesive within temporary wound dressings.^72^ Therefore, the mechanical performance of polyurethane 1 under different physiological conditions was investigated – films of polyurethane 1 were soaked in distilled water and a phosphate buffered saline (PBS) solution at both room temperature (19 ± 0.5 °C for 48 hours) and body temperature (37 °C for 12 hours). Mean stress–strain curves are shown in Fig. 6, and the corresponding mechanical properties calculated from the individual stress–strain curves (see Fig. S6†) are shown in Table 2. It was observed that both the modulus and strength decreased up to 30% after soaking in distilled water or PBS solution, but the elongation to break increased. This suggests that water diffuses into the polymer network and acts as a plasticiser, thus decreasing the stiffness and strength but improving failure resistance. Despite the water absorption, the chemical integrity of the bulk material is stable under exposure to these physiological conditions, as indicated by the mechanical performance of polyurethane 1 (compared to pristine polyurethane 1) at different physiological conditions (see Table 2).

**Table 2:** Mechanical properties from tensile testing of polyurethane 1 at different physiological conditions, means and (standard deviations) from at least five samples

Sample	Tensile modulus^a^ (MPa)	Yield strength (MPa)	Elongation at break (%)	Energy absorbed (MPa)
Pristine PU1	6.32 ± 0.36	0.68 ± 0.05	405 ± 47	207 ± 23
RT-DW	5.68 ± 0.21	0.58 ± 0.04	550 ± 140	194 ± 34
RT-PBS	4.61 ± 0.30	0.46 ± 0.02	535 ± 190	143 ± 35
37DEG-DW	5.09 ± 0.28	0.50 ± 0.03	473 ± 150	139 ± 40
37DEG-PBS	5.40 ± 0.65	0.53 ± 0.03	583 ± 160	182 ± 28

a This modulus was calculated using forces measured by the mechanical testing machine and local strains measured using an optical technique (digital image correlation) between strains of 0 and 3.5%. The value is therefore a linear approximation to the true, non-linear, polymer behaviour.

**Fig. 7:**
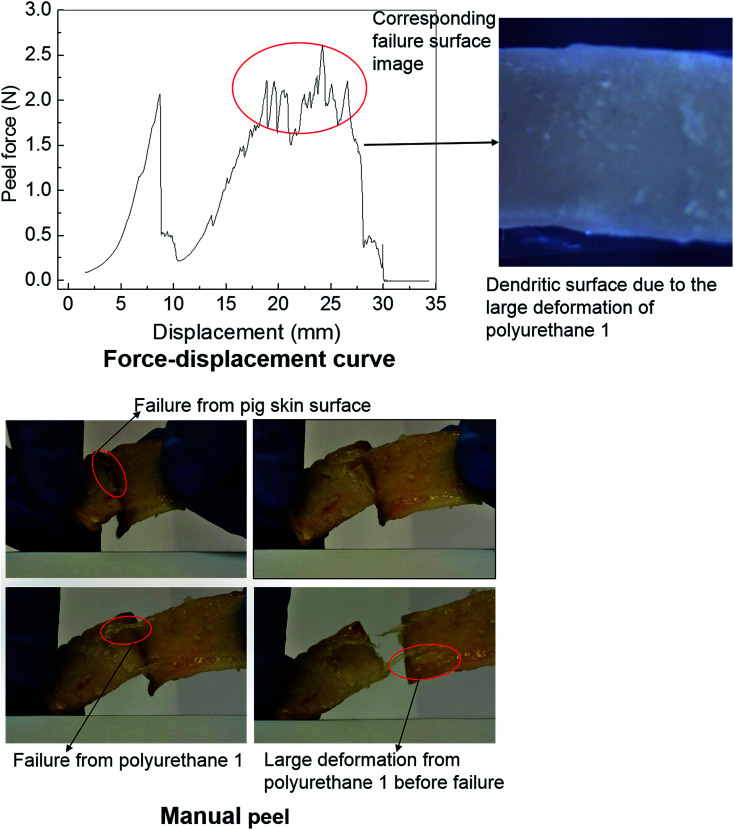
The investigation of the adhesive property of polyurethane 1 with a pig skin substrate.

Polyurethanes are routinely employed as adhesives in a diverse range of applications including biomedical devices.^26,29,67^ Within this context, the adhesive properties of polyurethane 1 were investigated using pig skin as a model substrate. A simple manual peel off test was carried out to investigate the adhesive properties of polyurethane 1 first. The skin was washed with acetone to remove residual fats and preservatives; a film of polyurethane 1 was then placed between two pieces of washed skin. The obtained sandwich structure with polyurethane 1 film in the middle was placed in an oven at a temperature of 37 °C for a period of 4 hours. Fig. 7 shows images of polyurethane 1 being removed from the skin manually. It can be observed that during the peel off, there is a large deformation of polyurethane 1 film, and that cohesive failure occurs occasionally (as shown in the pictures of the failure from the pig skin surface and the large deformation of polyurethane 1 before failure), which indicates that good bonding properties can be achieved between the pig skin and the film of polyurethane 1. To quantify the adhesive strength peel tests were performed on samples of width 1.25 mm and length 80 mm, using a commercial tensile test frame. A rig was designed to hold the sample and apply the loading. The specific arrangement of the test sample and setting of the rig is shown in Fig. S7 in the ESI.† As a result of the difficulties in cutting sufficiently flat skin samples, it proved impossible to maintain uniform contact (thus uniform pressure) across the whole sample during preparation, which leads to significant variation of the peel force during the test,^73^ as observed in Fig. 7 (force *vs.* displacement curve). However, preliminary results strongly suggest that stable peel strength can be generated and a peel force of 2 N can be achieved (the high force region corresponding to the dendritic failure surface due to the large deformation polyurethane 1 experienced during the peel test). In all, both the qualitative evidence (large plastic deformation of polyurethane 1 film and cohesive failure during the peel off) and quantitative data (peel strength) suggest good adhesive properties of polyurethane 1 to bind skin substrates.

**Fig. 8:**
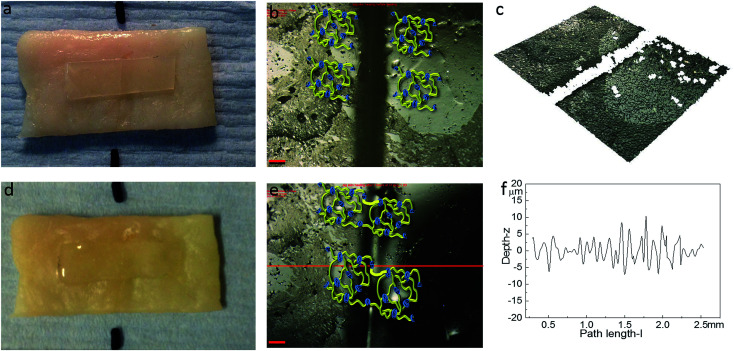
The healing of polyurethane on the surface of pig skin: (a) (overall view), (b) (2D microscopy), (c) (3D microscopy) corresponding to the sample before healing, and (d) (overall view), (e) (2D microscopy), (f) (surface roughness profile) corresponding to polyurethane 1 healing at the surface of pig skin after two hours. The surface roughness profile is from the middle of the whole sample across the crack horizontally as indicated in (e) by the red line. The scale bar in the pictures is 200 μm.

The healing capability of the adhered polyurethane 1 on the pig skin surface was also investigated. A sample was cut in the centre gently, transverse to its long axis, with a razor and positioned with the cut edges in close contact on the surface of pig skin. The sample was then placed in the oven at 37 °C for two hours. The images before healing and after healing for two hours were captured by high-resolution digital camera and optical surface profilometry, and the corresponding results are shown in Fig. 8. It was observed that a clear cut existed before healing, which disappeared completely after two hours healing at the temperature of 37 °C, as indicated by the surface roughness profile which shows that the roughness around the cut area is both qualitatively (*i.e.* visibly) and quantitatively comparable to the other areas in the surface of the sample, indicating a fully topological recovery of the cut interface. Therefore, the excellent healing capability of polyurethane 1 was maintained even when attached on the surface of pig skin.

**Fig. 9:**
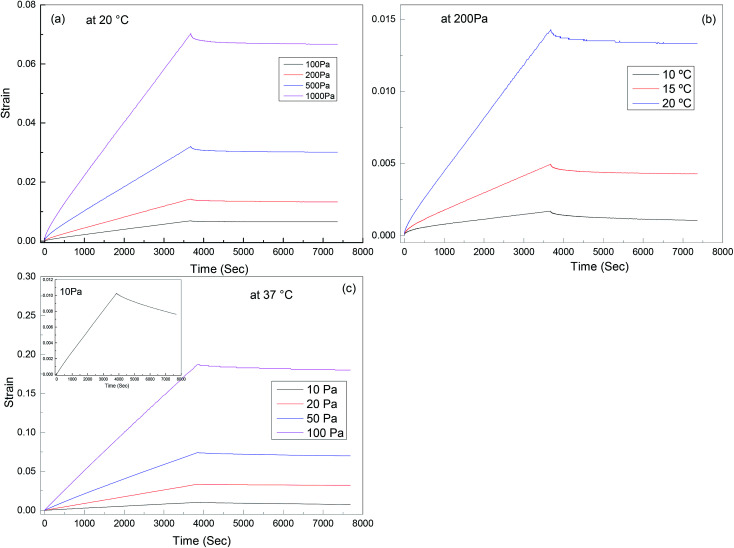
Creep-recovery behaviour of polyurethane 1: (a) stress dependence at 20 °C, (b) temperature dependence at 200 Pa; (c) stress dependence at 37 °C, inset shows recovery at 10 Pa in more detail.

Creep recovery experiments were performed in the same rheometer as described above to further characterise the viscoelastic response of the materials (see Fig. 9). It is observed that the creep behaviour of polyurethane 1 shows linear dependence on the stress level, but is very sensitive to temperature. For example, at 10 °C, 0.17% deformation is observed at a load of 200 Pa after about 1 hour, and 38% of this deformation can be recovered after 1 hour. This indicates good elasticity recovery of polyurethane 1, and is expected to be due to the strong non-covalent interaction between polymer chains from the hydrogen bonds. However, at a temperature of 20 °C, the deformation increases significantly to 1.43%, and only 5% of this deformation is recovered after 1 hour, which is consistent with the disruption of the hydrogen bonds at elevated temperatures; although this is not observed in the 5 Hz rheometer data (Fig. 2) until higher temperatures, it does have a significant effect on the creep and recovery behaviour on these longer timescales. The behaviour of polyurethane 1 is similar to another polyurethane we reported recently,^74^ but with a lower disruption temperature for the secondary interaction, which is consistent with the lower healing temperature (37 °C) of this material compared with 45 °C for that reported before, and also consistent with the observed recovery data for the two materials. Further creep recovery experiments were performed at 37 °C, the recovery at larger stresses was minimal, although at 10 Pa it was about 20%. It is anticipated that for biological applications the creep and recovery behaviour would be improved through the production of composite materials with suitable fillers.

**Fig. 10:**
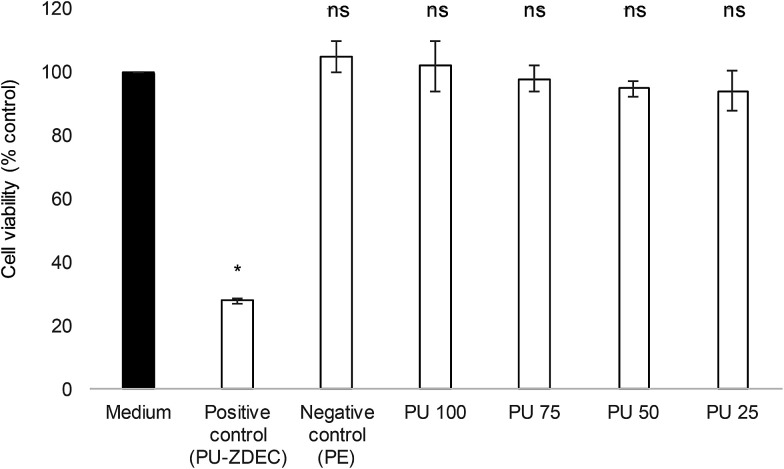
Cytotoxicity profile of liquid extracts from polyurethane at different concentrations (from 100%: PU100 to 25%: PU25). Polyethylene (PE) and polyurethane (PU) containing 0.1% (w/w) zinc diethyldithiocarbamate (ZDEC) were used as negative and positive controls, respectively. Data indicate average ± SEM, *n* = 3. Statistical significance with respect to untreated sample (medium) was determined by ANOVA followed by Bonferroni *post hoc* test and is indicated in the figure (* = *P* < 0.05; ns = non-significant).

Whenever a new material is suggested for therapeutic purposes, toxicity assessment is important to ensure that it is safe for use. Cytotoxicity studies were carried out on the human skin fibroblasts, 161BR cells by MTT assay. Polyurethane 1 was found to be non-toxic (cell viability after exposure to liquid extracts from the polymer >94% at all concentrations, and non-significantly different from the negative control, see Fig. 10).

## Conclusions

A well-defined supramolecular polyurethane capable of self-assembling *via* hydrogen bonding interactions has been synthesised. The material presents rheological behaviour characteristic of a supramolecular polymer, but with a low dissociation temperature for the network, which permits healing at 37 °C. Results show that after 60 minutes at body temperature, the material can fully recover its mechanical performance. In addition, the investigation of the mechanical performance under physiological conditions shows that the material can maintain its structural integrity. In addition, when adhered to pig skin, the healable properties of polyurethane 1 were fully conserved suggesting this material could be used for biomedical applications such as artificial skin or adhesives for plastic surgery.

## Supplementary Material

SC-007-C5SC04864H-s001
