# Supplementary material for: An adhesive elastomeric supramolecular polyurethane healable at body temperature
Source: Chem Sci. 2016 Mar 15;7(7):4291–300. doi: 10.1039/c5sc04864h (PMC6054028; doi:10.1039/c5sc04864h)
Supplement: SC-007-C5SC04864H-s001 [file SC-007-C5SC04864H-s001.pdf]

Electronic Supplementary Material (ESI) for Chemical Science.
This journal is © The Royal Society of Chemistry 2016

An adhesive elastomeric supramolecular polyurethane healable at body temperature

Antonio Feula,[a] Xuegang Tang,[b] Ioannis Giannakopoulos,[b] Ann M. Chippindale,[a] Ian W. Hamley,[a] Francesca Greco,[c] C. Paul Buckley,[b] Clive R. Siviour,[b] and Wayne Hayes[a*]

[a]Department of Chemistry, University of Reading, Whiteknights, Reading, RG6 6AD (UK); Fax: (+ 44) 118-378-6331; Email: w.c.hayes@reading.ac.uk

[b]Department of Engineering Science, Oxford University, Parks Road, Oxford, OX1 3PJ (UK)

[c]Reading School of Pharmacy, University of Reading, Whiteknights, Reading, RG6 6AD (UK)

Supplementary Information

Materials	**S-2**
Polyurethane synthesis	**S-2**
Methods	**S-3**
Sample preparation for Rheological and Mechanical Assessment	**S-4**
Mechanical testing	**S-4**
Cytotoxicity studies	**S-5**
**Figure S1.** ^{1}H NMR spectra of polyurethane **1** in $CDCl_3$ at 25 °C	**S-7**
**Figure S2.** ^{13}C NMR spectra of polyurethane **1** in $CDCl_3$ at 25 °C	**S-7**
**Figure S3.** DSC thermogram of polyurethane **1**	**S-8**
**Figure S4.** Frequency sweep for supramolecular polyurethane **1** at different temperatures	**S-9**
**Figure S5.** The stress-strain curves for pristine and healed polyurethane **1**	**S-10**
**Figure S6.** The stress-strain curves for pristine polyurethane **1** under different physiological conditions	**S-11**
**Figure S7.** The schematic of sample mounting and peel test loading rig for pig skin peel test	**S-12**
**Reference**	**S-13**

Materials

All chemicals and solvents were purchased from Sigma Aldrich and used as received. Krasol HLBH-P2000 was supplied by Cray Valley.

Polyurethane synthesis

Methylene diphenyl diisocyanate (25.00 mmol, 6.25 g) was added to Krasol™ HLBH-P2000 (11.90 mmol, 25 g) and the mixture was stirred for three hours at 80 °C under a nitrogen atmosphere after which dry THF (80 mL) was added. The reaction was left to cool to room temperature, 4-(2-aminoethyl)morpholine (26.18 mmol, 3.43 g) was then added and the mixture was stirred for one hour at 50 °C. THF was then removed *in vacuo* and the product dissolved in the minimum volume of $CHCl_3$ before purification by precipitation in methanol before drying *in vacuo*. The product was isolated as a pink coloured solid in 93% yield, 31.67 g. ATR FTIR (Neat) ν 1221, 1309, 1378, 1412, 1460, 1537, 1598, 1658, 1706, 2851, 2919, 2958, 3324 cm^{-1}; ^{1}H NMR (400 MHz; $CDCl_3$) δ_H ppm 0.81-1.69 (388H, m), 2.44 (8H, m, 4 x N**CH_2**CH_2O), 2.50 (4H, t, *J* = 6.0, 2 x NHCH_2**CH_2**N), 3.33 (4H, q, *J* = 5.6, 2 x NH**CH_2**CH_2N), 3.63-3.64 (8H, m, 4 x NCH_2**CH_2**O), 3.88 (4H, s, 2 x Ar**CH_2**Ar), 4.12-4.19 (4H, m, 2 x **CH_2**OC=O), 5.31-5.41 (2H, br, 2 x N**H**), 6.51-6.63 (2H, br, 2 x N**H**), 7.09-7.29 (18H, m, Ar**H**); ^{13}C NMR (100 MHz; $CDCl_3$) δ_C ppm 10.8, 25.9, 26.8, 29.8, 30.2, 30.7, 33.2, 33.4, 36.1, 38.9, 40.5, 50.4, 53.4, 57.8, 63.8, 65.3, 118.9, 121.5, 129.3, 129.4, 129.4, 136.1, 136.6, 136.7, 137.0, 153.8, 156.2; GPC (THF), Mw 4287, Mn 4097, Đ 1.046.

Methods

^{1}H NMR and ^{13}C NMR spectra were recorded on either a Bruker Nanobay 400 or a Bruker DPX 400 spectrometer operating at 400 MHz for ^{1}H NMR or 100 MHz for ^{13}C NMR. The samples for NMR spectroscopic analysis were prepared in $CDCl_3$ (~50 mg/mL) and dissolution was aided with slight heating. The data was processed using MestReNova Version 7.0.2-8636. Chemical shifts (δ) are reported in ppm relative to tetramethylsilane (δ 0.00 ppm) for ^{1}H NMR spectra and to chloroform (δ 77.0 ppm) for the ^{13}C NMR assignment, coupling constants (*J*) are expressed in Hertz (Hz). Infrared spectroscopic analysis employed a Perkin Elmer 100 FTIR spectrometer equipped with

a diamond ATR sampling attachment and samples were analysed in neat form. The infrared spectroscopic data was processed using Microsoft Excel 2013. Gel permeation chromatography (GPC) was conducted using an Agilent Technologies 1260 Infinity and the data was processed using Agilent GPC/SEC software, polystyrene was used as the calibrant. Samples for GPC analysis were dissolved in analytical grade THF (2 mg/mL). Differential scanning calorimetric analysis used a TA Instruments DSC 2920 and a three cycle process was carried out on the solid sample; heating from ambient to +100 ºC at a ramp rate of 10 ºC min^{-1}, followed by cooling from +100 ºC to -70 ºC at a ramp rate of -20 ºC min^{-1} and then finally by heating from -70 ºC to +200 ºC at a ramp rate of +20 ºC min^{-1}. The sample size used was 8-15 mg and the data was processed using TA Universal Analysis Version 4.7A. Thermogravimetric analysis employed a TGA Q50 instrument by heating the solid samples (sample size *ca*. 20 mg) from ambient temperature to +300 ºC at a ramp rate of +10 ºC min^{-1}. The data was processed using TA Universal Analysis Version 4.7A. SAXS/WAXS was performed using a Bruker Nanostar system. The SAXS data were recorded on a Vantec detector and the WAXS data were recorded using a FujiFilm image plate system. Samples were mounted in a temperature-controlled heater, in the form of films sandwiched between Kapton films. The SAXS camera length was 66 cm and the q = 2*pi*sin(theta)/lambda (scattering angle = 2*theta, lambda = 1.54 Angstrom) scale was calibrated using silver behenate, the d-spacing is 58.3 Å. The WAXS IP is a Fujifilm imaging plate (BAS-IP MS 2025), which is read on a Fujifilm FLA-7000 and wiped clean on a Fujifilm IP Eraser 3. The sample to detector distance is 55 mm. The calibrant for WAXS is corundum, which has a d-spacing of 2.55Å. Single crystal X-ray was performed on Agilent Gemini S Ultra dual Cu and Mo radiation source, Cu radiation at 1.54Å, Mo at 0.71Å with a CCD Sapphire detector. Data collection was carried out at 150 K.

Sample preparation for rheological and mechanical assessment

Thin films of the polyurethane were produced for mechanical testing via a solution casting procedure. The polyurethane was dissolved in THF and the solution was poured into flat PTFE molds. This was subsequently placed in a vacuum oven at 70 ºC with a pressure of approximately 0.6 to 0.8 bar for a duration of 16 hours. Polyurethane film of uniform thickness between 200 and 500 μm was obtained at the end of this procedure

without residual solvent. Rheological analysis was performed on circular samples (25 mm diameter) that were obtained from the cast film using a steel punch cutter. For tensile testing, rectangular samples with length 40 mm, width 5 mm and thickness 0.5 mm were cut with a razor blade and paper end-tabs were bonded to the samples with a commercial cyanoacrylate adhesive Loctite™. This sample assembly was found to reduce slippage inside the tensile grips of the tensometer. The initial gauge length was 25 mm. A Peltier heating system was used to maintain the temperature of 37 ºC during the healing process for image observation by microscopy, the healed samples for mechanical testing were prepared in an oven at 37 ºC.

Mechanical testing

Parallel plate oscillatory shear was performed with an Anton Paar Physica MCR 301 rheometer. For the temperature sweep at a single frequency of 5 Hz, the samples were placed on the rheometer, heated to 70 ºC and held at this temperature for approximately 5 minutes. The samples were then subjected to a cooling temperature ramp of 2 ºC/min to 0 ºC before being heated up to 120 ºC and held at this temperature for 1 hour. After this step they were cooled again from 120 ºC to 0 ºC at 2 ºC/min. During all of these steps the dynamic shear moduli (G', G" and tanδ) of the samples were recorded. This method was applied to ensure that chemical cross-linking had not occurred from the exposure of the samples to high temperatures. Cross-linking was not observed during rheological testing, i.e. an increase in G' during the isothermal step at 120 ºC was not evident. The samples were tested at a strain amplitude of 0.1%.

For the isothermal frequency sweeps, the samples were again placed in the rheometer and heated to 70 ºC for approximately 5 minutes. The temperature was reduced to 20 ºC, and again held for 5 minutes. The frequency sweeps were the performed between 0.1 and 100 rad/s at a strain amplitude of 0.1% for each selected temperature, each time the temperature was held for 5 minutes before data were gathered. Three cycles of tests were measured, and the results show that the repeating heating does not change the properties significantly. Creep tests were also carried out at different temperatures and stress levels using the MCR 301 rheometer. To achieve good bonding between the plate and material, the sample was treated at the temperature of 80 ºC when in contact with

the plate for a period 10 minutes before cooling to room temperature and subsequent analysis.

The tensile testing was carried out on an Instron universal testing machine (model 5982) with a 100 N load cell. A constant true strain rate of 0.04 s^{-1} was used, for purposes of controlling the experiment (but not subsequent data analysis) this calculated from the cross-head displacement. At least five samples were tested for each material type. In order to obtain accurate, and full-field displacement and strain data, the Digital Image Correlation technique was used. The surface of the sample was painted with both white and black sprays in order to create a random speckle pattern. During each test, a digital camera (Point Gray GS3-U3-41C6C-C with a Micro-NIKKOR 105 mm lens), was used to record images of the specimen surface at a rate of 5 pictures per second. Hundreds of images were thus created before specimen failure. The obtained images were analysed using commercially available image correlation software (DaVis) to extract displacement and strain maps on the specimen surface. The mean strain was combined with force data from the Instron testing machine to generate stress-strain curves. Furthermore, the strain fields were used to examine the possibility of strain localisation around the healed region: none was observed.

For pig skin peel tests, the polyurethane **1** film was first sandwiched between two pieces of pig skin, and then the sandwiched sample (with width 1.25 mm and length 80 mm) was placed in an oven at a temperature of 37 ºC for 4 hours. The sample thus obtained was glued onto an aluminium plate, and a polyester film was further glued onto the back of the top pig skin surface. This film was clamped in the loading system. The aluminium plate was attached to a support, which is free to move along the direction of the peel, and the test was then performed by lifting the polyester film using a commercial mechanical test frame. The test rate was controlled by the load frame, and the test was carried out at room temperature.

Cytotoxicity studies

In vitro cell culture studies were conducted using a human skin fibroblast cell line (161BR, ECACC). Cells were cultured in EMEM (EBSS) with 2 mM glutamine medium supplemented with 15% foetal bovine serum (FBS), 1% non-essential amino acids (NEAA), penicillin (100 units/mL) and streptomycin (0.1 mg/mL). Cells were

maintained in a humidified atmosphere at 37 °C and 5% CO_2. Potential *in vitro* cytotoxicity effects of the polyurethane were assessed as follows. Liquid extracts of the samples were prepared by exposing the polymeric film to complete medium (3 cm^2/mL, for 24 hours) with polyethylene (PE) and polyurethane (PU) containing 0.1% (w/w) zinc diethyldithiocarbamate (ZDEC) used as negative and positive controls, respectively (the latter materials were supplied by Hatano Research Institute, Food and Drug Safety Center). These liquid extracts were filter sterilised and diluted (100%, 75%, 50% and 25%) prior to application onto the cells.

Cells were seeded in a 96 well plate at 4×10^4 cells/mL in complete medium and allowed to adhere for 24 hours. Then, the medium was removed and replaced with either: (a) control medium, (b) liquid extracts from the negative control (PE), (c) liquid extract from the positive control (PU-ZDEC), or (d) liquid extracts from the test polymer at the various dilutions, and cells incubated for a further 67 hours, after which an MTT assay was performed.[1]

Briefly, MTT solution (5 mg/mL in PBS) was added to each well (20 μl/well). After 5 hours (i.e. at 72 hours) the solution was removed and replaced with DMSO (100 μl/well), to dissolve the formazan crystals. The plate was then analysed at a UV microplate reader (λ = 570 nm). Results were reported as % cell viability compared to the untreated control (medium).

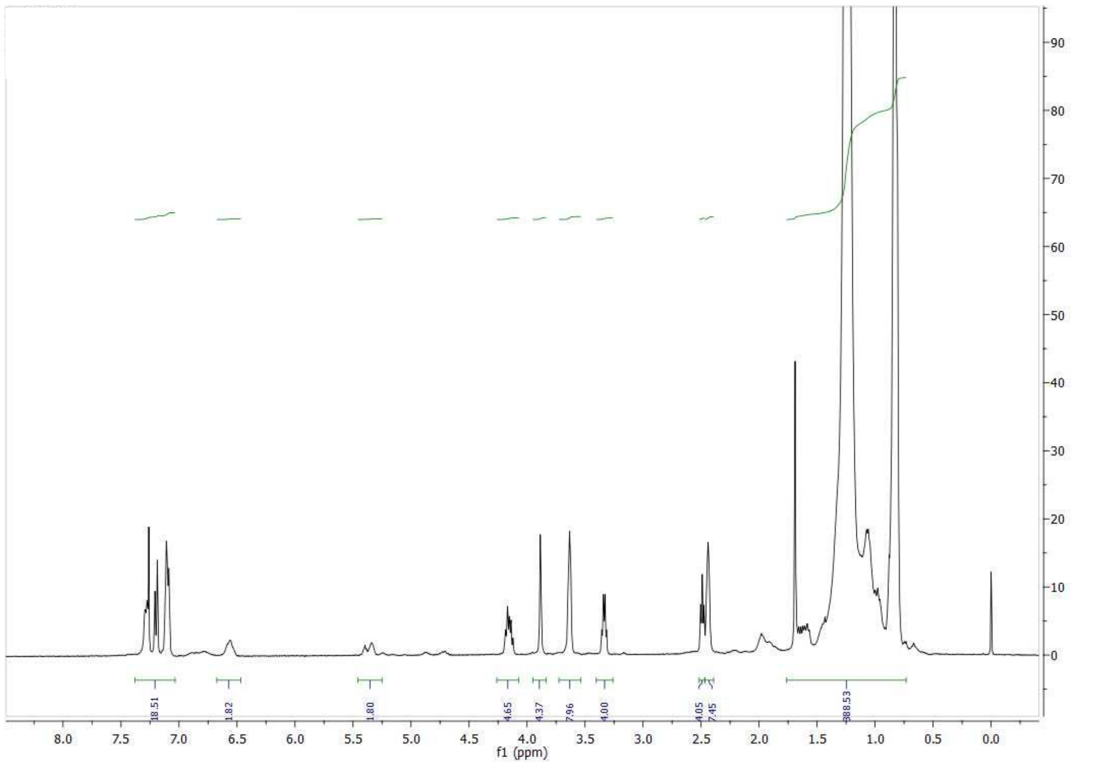

Figure S1. ^{1}H NMR of polyurethane **1** in $CDCl_3$ at 25 °C

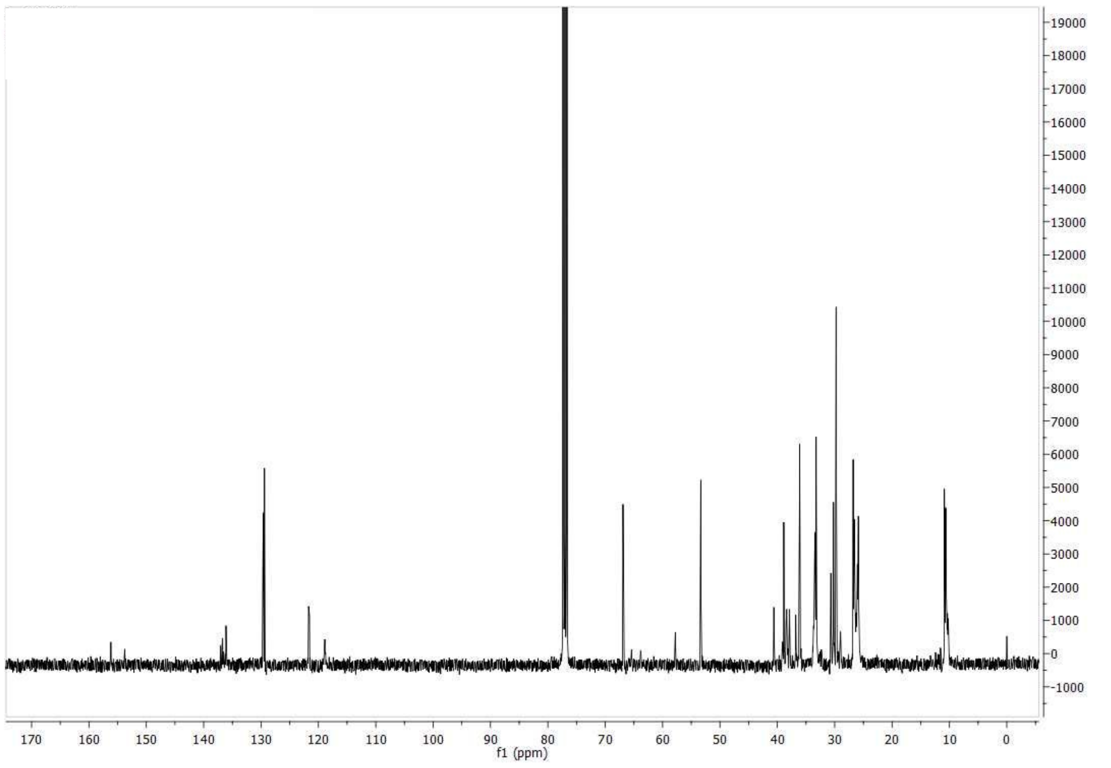

Figure S2. ^{13}C NMR of polyurethane **1** in $CDCl_3$ at 25 °C

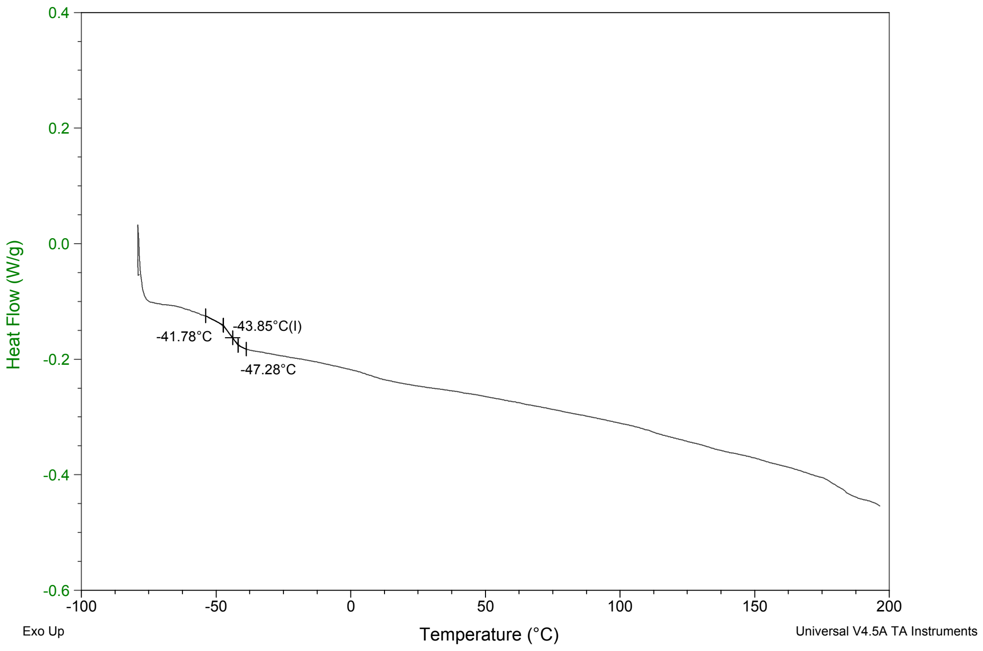

Figure S3. DSC thermogram of polyurethane **1**

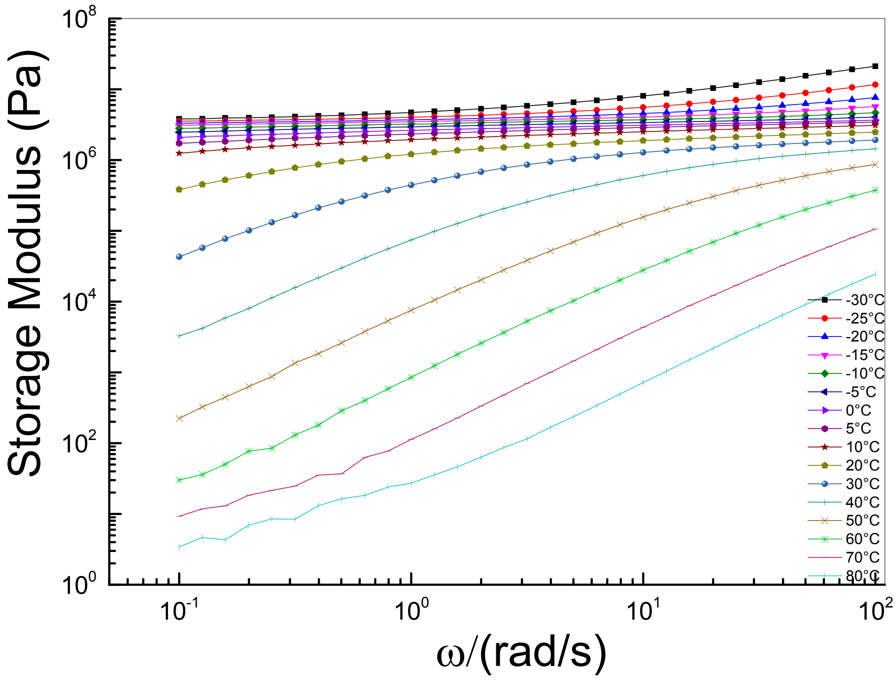

Figure S4. Frequency sweep for supramolecular polyurethane **1** at different temperatures. Isothermal frequency sweep data obtained at frequencies between 0.1 and 100 rad s^{-1} and at 5 ºC intervals between -30 and 10 ºC and 10 ºC intervals between 10 and +80 ºC.

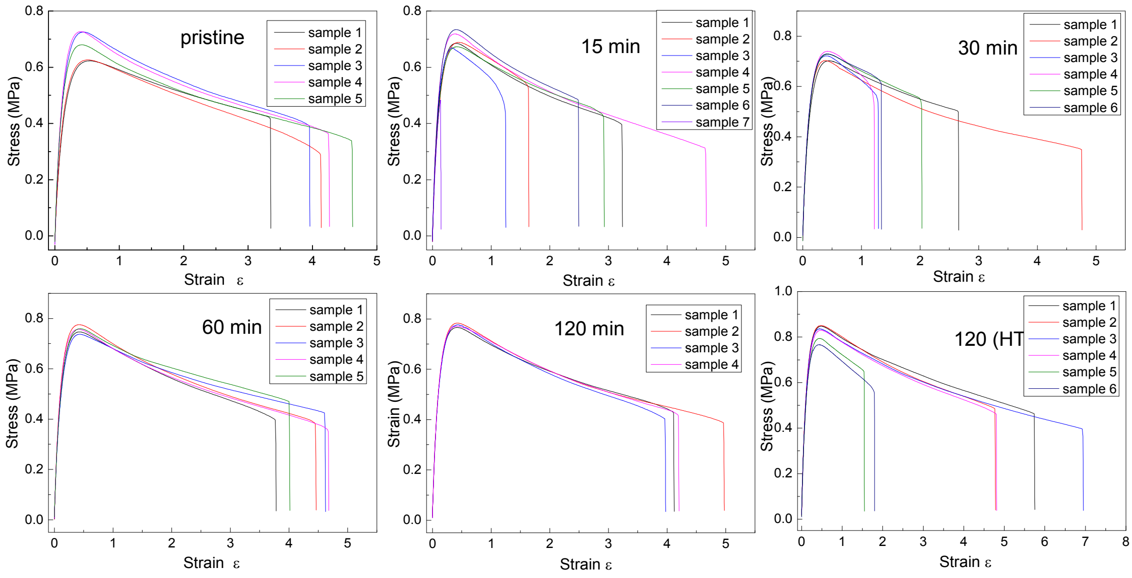

Figure S5. The stress-strain curves for pristine and healed polyurethane **1**. Pristine) pristine polyurethane **1** without any heat treatment, 15 min) healing after 15 minutes, 30 min) healing after 30 minutes, 60 min) healing after 60 minutes, 120 min) healing after 120 minutes, 120 min (HT)) heat treated pristine polyurethane **1** at 37 ºC for 120 minutes. The ends of the curves represent specimen failure.

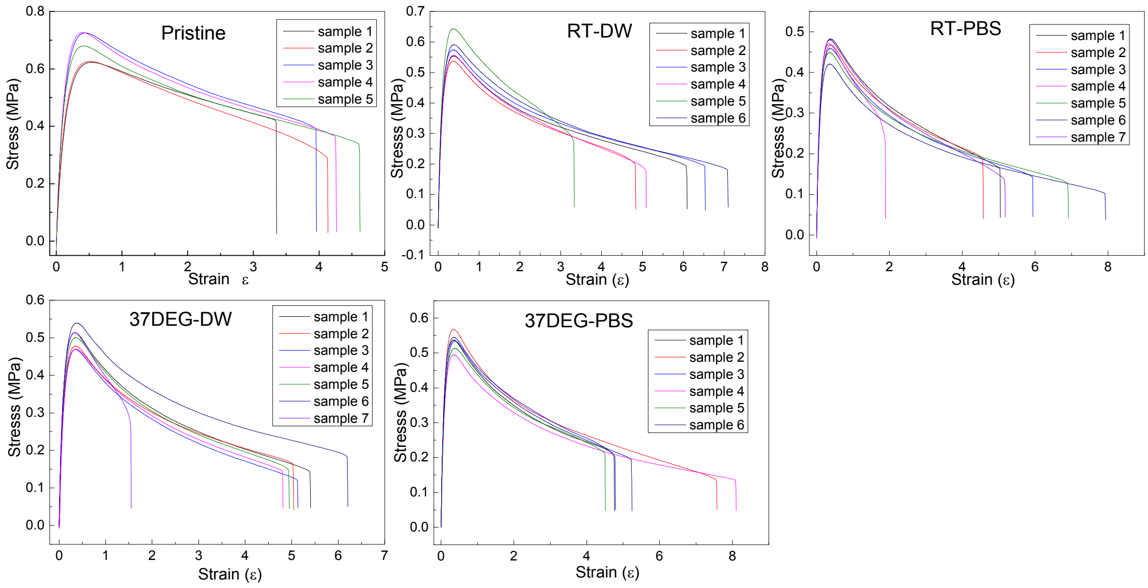

Figure S6. The stress-strain curves for pristine polyurethane **1** under different physiological conditions. Pristine) pristine polyurethane **1**, RT-DW) room temperature in distilled water for 48 hours, RT-PBS) room temperature in PBS solution for 48 hours, 37 DEG-DW) 37 ºC in distilled water for 12 hours, 37 DEG-PBS) 37 ºC in PBS solution for 12 hours. The ends of the curves represent specimen failure.

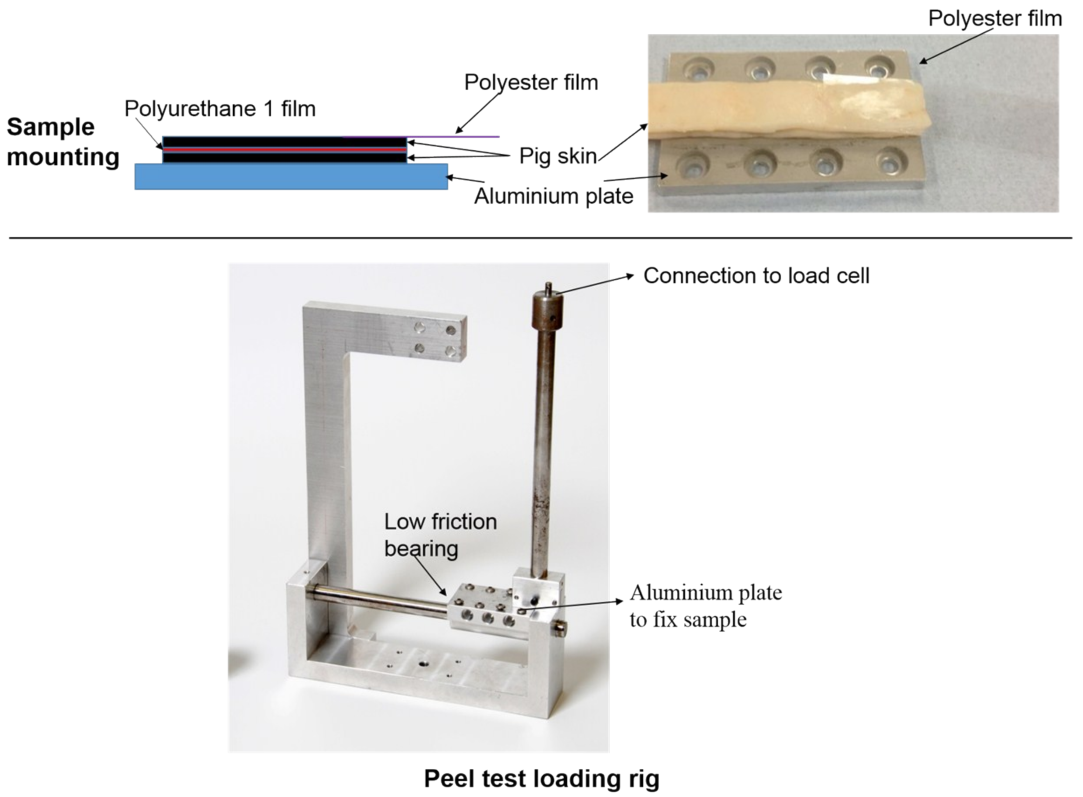

Figure S7. A schematic representation of sample mounting and peel test loading rig for the pig skin peel test.

Reference

1. F. Greco, I. Arif , R. Botting, C. Fante, L. Quintieri, C. Clementi, O. Schiavon, G. Pasut, *Polym. Chem.*, 2013, **4**, 1600-1609.
